# Therapeutic effects of whole-body vibration on postmenopausal women with osteoporosis: a systematic review and meta-analysis

**DOI:** 10.1590/1414-431X2024e13996

**Published:** 2024-11-04

**Authors:** Qian Li, Lichao Liang, Chengfei Gao, Beibei Zong

**Affiliations:** 1 Department of Rehabilitation Medicine, Zibo Central Hospital, Zibo, Shandong, China; 2 Department of Rehabilitation Medicine, the Affiliated Hospital of Qingdao University, Qingdao, Shandong, China

**Keywords:** Whole-body vibration, Postmenopausal osteoporosis, Bone mineral density, Meta-analysis, Randomized controlled trails

## Abstract

The objective of this study was to assess the efficacy of whole-body vibration (WBV) on bone mineral density (BMD), pain levels, and body composition in postmenopausal women with osteoporosis (PMOP). Relevant studies were retrieved from the PubMed, EMBASE, Web of science, CENTRAL, and PEDro databases. Thirteen randomized controlled trials with 783 patients were enrolled. The meta-analysis results showed that WBV can significantly increase lumbar spine BMD (WMD=0.018; 95%CI: 0.004 to 0.032; P=0.011), femoral neck BMD (WMD=0.005, 95%CI: 0.001 to 0.011, P=0.0493), and reduce pain degree (WMD=-0.786; 95%CI: -1.300 to -0.272; P=0.0027) in PMOP, but has no significant effect on patients' muscle mass (WMD=0.547; 95%CI: -1.104 to 2.199; P=0.5158) as well as fat mass (WMD=0.530; 95%CI: -2.389 to 3.448; P=0.7222). To conclude, WBV showed the potential to provide positive benefits in improving BMD and relieving pain of PMOP.

## Introduction

Osteoporosis is a metabolic bone disease that manifests as decreased bone strength, deteriorated bone microarchitecture, and increased susceptibility to fragility fractures (1). Osteoporosis occurs primarily in postmenopausal women caused by the deficiency of estrogen, which leads to an imbalance between bone resorption and formation (2). Approximately half of postmenopausal women will experience fragility fractures, leading to pain, disability, and impaired quality of life (3).

The management of postmenopausal osteoporosis (PMOP) can be broadly classified into general management and pharmacologic interventions aimed at inhibiting bone resorption or promoting bone formation (4,5). However, the use of drugs may produce side effects, including gastrointestinal reactions, renal toxicity, osteonecrosis of the jaw and so on, which worsen patient compliance despite their effectiveness (5,6). In addition to antiosteoporotic drugs, multicomponent exercise programs, such as weight-bearing and resistance exercises, have gained significance in the treatment of osteoporosis in recent years. However, this approach has the inherent disadvantage of lacking long-term compliance and may even increase the risk of fractures (7). Whole-body vibration (WBV), a safe and acceptable exercise program performed with the body on a vibration platform, has demonstrated the potential to enhance bone mass in experimental models of ovariectomized rats (8,9). It has been demonstrated that WBV can induce osteogenic effects by increasing blood flow within bones via direct bone stimulation and mechanotransduction. Alternatively, WBV can provide indirect bone stimulation through the activation of skeletal muscles via tonic vibration reflex (10,11).

Clinical studies also confirmed the anabolic effects of WBV on the skeleton by activation of the musculature through mechanical transduction of the vibration strain within the bone (12,13), thereby offering novel perspectives for osteoporosis prevention and treatment. It is known that hormonal changes, particularly the reduction in estrogen, significantly increases the risk of osteoporosis. WBV may help mitigate these effects by improving bone mineral density and affecting serum levels of anabolic hormones in postmenopausal women (14). Nevertheless, due to variations in study protocols, such as vibration parameters, disease duration, and treatment duration, the efficacy of WBV therapy in PMOP is still controversial (15,16). The aim of this study was to perform a comprehensive quantitative analysis of published randomized controlled trails (RCTs) to explore the efficacy of WBV on bone mineral density (BMD), pain levels, and body composition in postmenopausal women with osteoporosis. The results will provide robust evidence-based references for application of WBV in the management of postmenopausal osteoporosis.

## Methodology

This study followed the Preferred Reporting Items for Systematic Reviews and Meta-Analyses (PRISMA) guidelines (17). The protocol has been registered in PROSPERO with number CRD42024504284.

### Eligibility criteria

We collected all available randomized controlled trials assessing the efficacy of WBV on either BMD (lumbar spine, femoral neck), pain, or body composition (fat mass and muscle mass) in postmenopausal women with osteoporosis. According to the World Health Organization definition, a t-score of less than 2.5 SD is diagnosed as osteoporosis (18). Studies were excluded if they compared different WBV protocols or had incomplete data that could not be obtained from the authors. Retrospective trials, case reports, and conference papers were also excluded.

### Search strategy

Potentially relevant studies were retrieved from the electronic databases of PubMed, Web of Science, EMBASE, CENTRAL, and PEDro from inception to July 1, 2024 with no language restrictions. Search terms and their combinations used in the search strategy were as follows: “WBV”, “whole body vibration”, “vibration training”, “PMOP”, “postmenopausal”, “osteoporosis”, “postmenopausal osteoporosis”. Reference lists from relevant studies were manually scanned for additional records. All retrieved records were imported into EndNote where duplicates were removed.

### Study selection and data extraction

Two reviewers (Q.L. and L.C.L.) independently screened the titles/abstracts to identify the relevant studies, and the full text was subsequently assessed to identify the eligibility for data extraction. Any disagreement between reviewers was resolved through consensus with a third reviewer (C.F.G).

The following information was extracted and recorded: first author, publication year, study design, patient age, intervention and control group information, outcome measures, and follow-up period. The assessment of bias risk for the included studies was conducted using the PEDro criterion (19).

### Data synthesis and analysis

We pooled the weighted mean difference (WMD) to calculate effect sizes, given that the different studies reported statistics and variances with the same units. Heterogeneity among included studies was evaluated with the I^2^ statistic, and I^2^>50% was considered as high heterogeneity (20). A fixed-effect model was applied when there was no significant heterogeneity between studies; otherwise, a random-effect model was performed. Egger's test and funnel plot were used to examine potential publication bias (21). A P-value <0.05 was considered statistically significant. The analyses were performed with Stata software 16.0 (StataCorp LP, USA).

## Results

### Search results and characteristics of included studies

**Figure 1:**
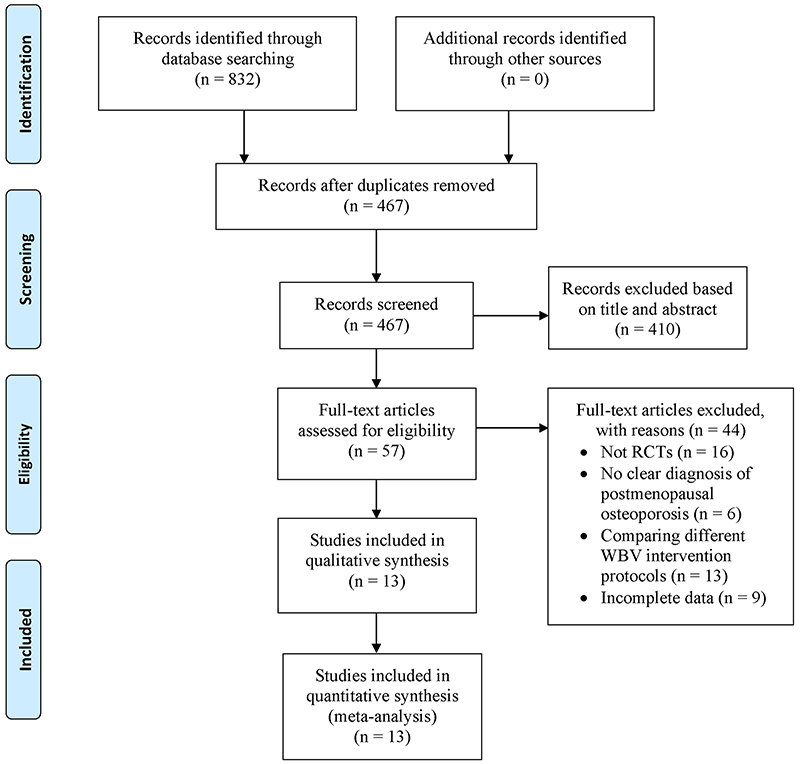
PRISMA flow diagram of study selection.

A total of 832 studies were initially retrieved from database searches. After duplicated records were removed, 467 titles and abstracts were reviewed and filtered for inclusion. Following the filtering process, 57 full-text articles were screened for eligibility. Forty-four studies were further excluded, including 16 non-randomized controlled studies, 6 studies without clear diagnosis of postmenopausal osteoporosis, 13 studies comparing different WBV intervention protocols, and 9 studies with incomplete data. Finally, 13 RCTs were included for data extraction and final meta-analysis. Figure 1 illustrates the study selection flow.

Thirteen RCTs with 783 participants were included in this meta-analysis, and the studies were conducted between 2004 and 2020. Regarding the WBV parameters, the frequency ranged from 12.5-90 Hz, and the magnitude ranged from 0.3 to 8 g. The weekly frequency of WBV ranged from one to seven times, and the exposure time between 4 to 30 min. All 13 RCTs evaluated BMD at the lumbar spine and femoral neck regions. Three studies evaluated body composition, and only 2 studies assessed pain score. The length of follow-up within the studies was 6 months to one year. The PEDro scale ranged from 5-9 points with an average of 7.2 points, showing that the included studies were of good methodological quality. The detailed study characteristics are summarized in Table 1 (15,22-33).

**Table 1:** Characteristics of eligible studies.

Author, Year	Study design	Study group (n)	Age (Mean±SD)	Intervention	Outcomes	Follow-up (month)	PEDro score
Verschueren et al. 15 2004	RCT	WBV^25^	64.6±3.3	WBV protocol: 35-40 Hz, 2.28-5.09 g, 30 min/session,3 times/wk	Femoral neck BMD, lumbar BMD, fat mass, muscle mass	6	8
		Control 25	64.2±3.1	Not participate in any training			
Lai et al. 22 2013	RCT	WBV^16^	60.1±7.1	WBV protocol: 30 Hz, 3.2 g, 5 min/session, 3 times/wk		6	6
		Control 16	62.4±7.1	Maintain daily life habits			
ElDeeb et al. 23 2020	RCT	WBV^25^	55.1±4.2	WBV protocol: 20-35 Hz, 5-10 min/session, 2 times/wk	Lumbar BMD, femoral neck BMD	6	7
		Control 25	57.3±4.4	Receive calcium and vitamin D supplementations			
Iwamoto et al. 24 2005	RCT	WBV^25^	71.9±8.1	WBV protocol: 20 Hz, 4 min/session, 1 time/wk+Alendronate 5 mg/day	Lumbar BMD, pain	6 and 12	7
		Control 25	70.6±8.7	Alendronate 5 mg/day			
Von Stengel et al. 25 2011	RCT	WBV63	68.0±3.9	WBV protocol: 12.5/35 Hz, 8 g, 15 min/session, 3 times/wk	Femoral neck BMD, lumbar BMD	12	10
		Control 33	67.6±4.1	Maintain light physical exercises and relaxation exercises			
Sen et al. 26 2020	RCT	WBV^15^	55.0±4.6	WBV protocol: 30-35 Hz, 10 min/session, 1-2 times/wk	Femoral neck BMD, lumbar BMD, hip BMD	6	6
		Control 18	54.5±6.0	Maintain routine daily life activities			
Jepsen et al. 27 2019	RCT	WBV^15^	55.0±4.6	WBV protocol: 30 Hz, 3.6 g, 6 min/session, 3 times/wk+Teriparatide: 20 μg/day	Lumbar BMD, hip BMD	6 and 12	8
		Control 18	54.5±6.0	Teriparatide 20 μg/day			
Ruan et al. 28 2008	RCT	WBV 51	61.2±8.2	WBV protocol: 30 Hz, 10 min/session, 5 times/wk	Femoral neck BMD, pain	6	7
		Control 43	63.7±5.4	Without any treatment			
Slatkovska et al. 29 2011	RCT	WBV135	60.1±6.5	WBV protocol: 30/90 Hz, 0.3 g, 20/session, 7 times/wk	Femoral neck BMD, lumbar BMD, hip BMD	12	9
		Control 67	60.8±5.5	Receive calcium and vitamin D supplementations			
Marín-Cascales et al. 30 2017	RCT	WBV^25^	59.6±5.9	WBV protocol: 35-40 Hz, 10 min/session, 3 times/wk	Lumbar BMD, fat mass, muscle mass	6	5
		Control 15	62.4±5.1	Maintain normal daily routines and dietary intake			
Tankisheva et al. 31 2015	RCT	WBV^15^	75.7±6.6	WBV protocol: 25-45 Hz, 1.71-3.58 g, 3 times/wk	Hip BMD, fat mass, muscle mass	6	7
		Control 16	77.6±6.8	Not change lifestyle			
de Oliveira et al. 32 2019	RCT	WBV^17^	56.4±6.5	WBV protocol: 20 Hz, 3.2 g, 5 min/session, 3 times/wk	Femoral neck BMD, lumbar BMD, hip BMD	6	9
		Control 17	54.1±5.3	Not carry out any type of intervention			
Santin-Medeiros et al. 33 2015	RCT	WBV^19^	82.3±5.1	WBV protocol: 20 Hz, 6 min/session, 2 times/wk	Femoral neck BMD, hip BMD	6	5
		Control 19	82.2±6.4	Not participate in any training program			

RCT: randomized controlled trial; BMD: bone mineral density; WBV: whole-body vibration.

### Outcomes

**Figure 2:**
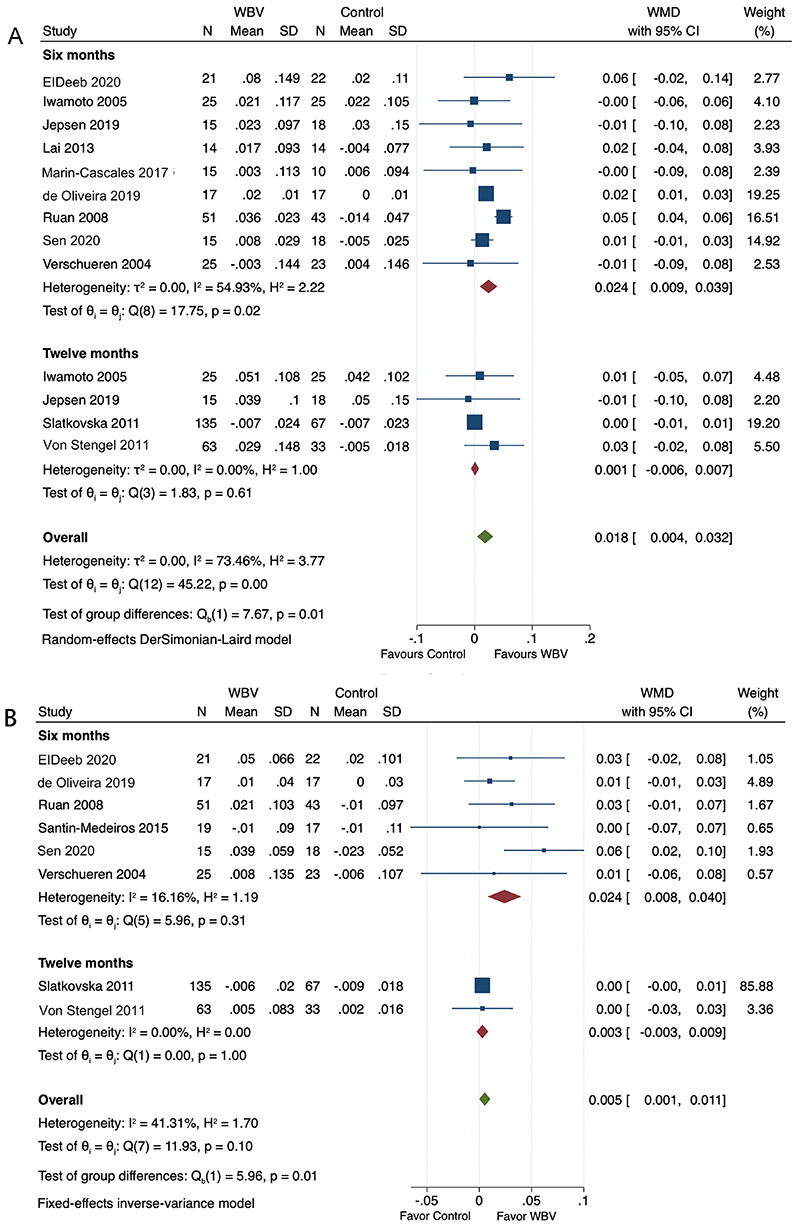
Forest plots of whole-body vibration (WBV) compared to control on lumbar spine bone mineral density (BMD) (**A**) and femoral neck BMD (**B**).

In total, 11 of 13 studies reported the efficacy of WBV on lumbar spine BMD of postmenopausal women with osteoporosis. The pooled analysis detected a significantly higher BMD in the WBV group compared with the control group (WMD=0.018; 95%CI: 0.004 to 0.032; P=0.011). Further subgroup analysis stratified by follow-up interval demonstrated a significant difference in lumbar spine BMD changes between the two groups at 6-month follow-up (WMD=0.024; 95%CI: 0.009 to 0.039; P=0.002), while no significant difference was observed at 12-month follow-up (WMD=0.001; 95%CI: -0.006 to 0.007; P=0.85; Figure 2A).

Eight RCTs analyzed the efficacy of WBV on femoral neck BMD of patients with osteoporosis, and pooled analysis revealed the WBV group had a significant impact in femoral neck BMD compared with the control group (WMD=0.005; 95%CI: 0.001 to 0.011; P=0.0493). In the subgroup analyses, the WBV group showed a significant increase in femoral neck BMD compared to the control group at 6-month follow-up (WMD=0.024; 95%CI: 0.008 to 0.040; P=0.0032), while no significant difference between the two groups was found at 12-month follow-up (WMD=0.003; 95%CI: -0.003 to 0.009; P=0.2906; Figure 2B).

**Figure 3:**
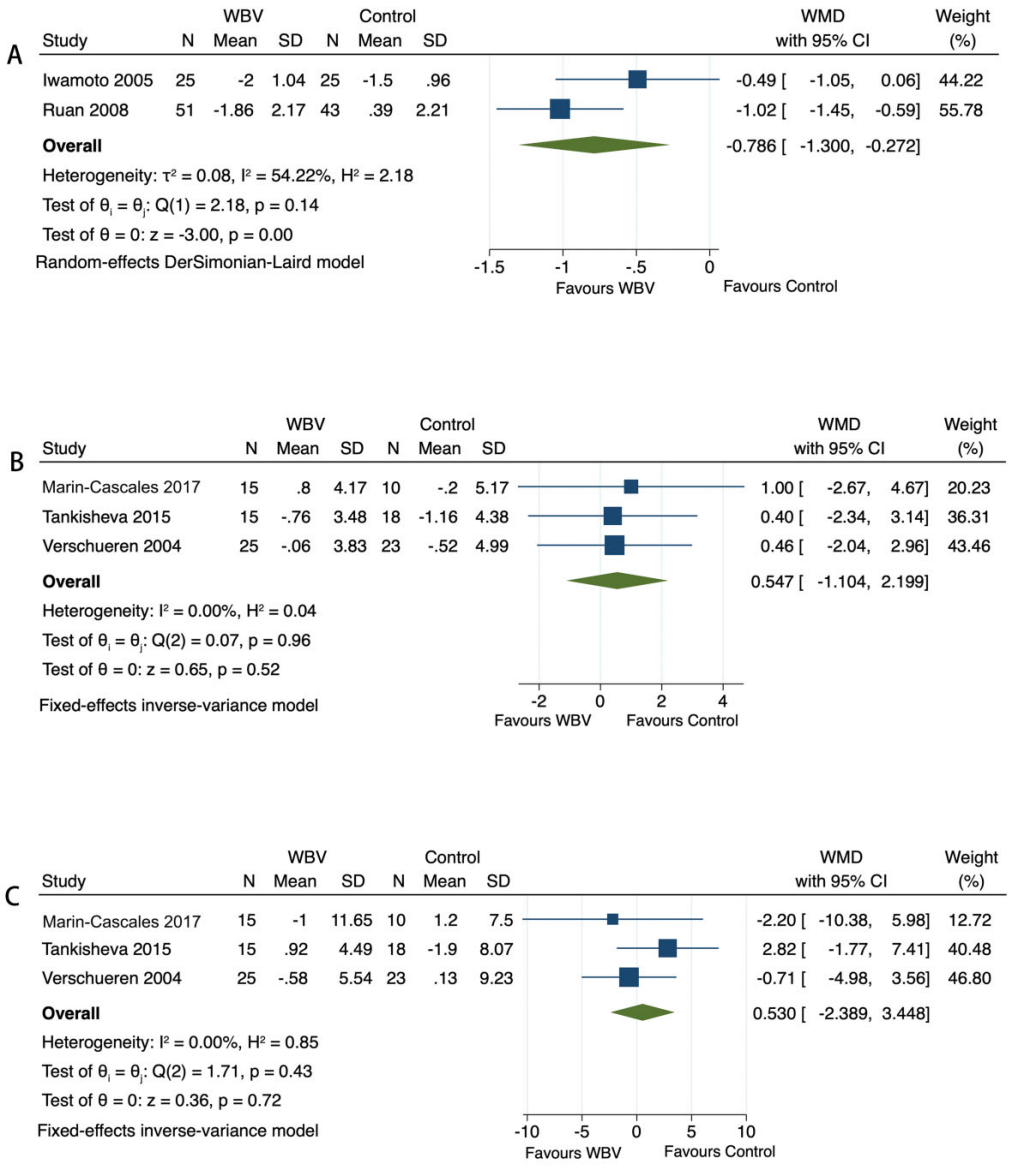
Forest plots of the whole-body vibration (WBV) group compared to the control group on pain (**A**), muscle mass (**B**), and fat mass (**C**).

Only two studies assessed the pain score after intervention, and pooled analysis detected a statistically significant decreased visual analogue scale (VAS) score in the WBV group compared to the control group (WMD=-0.786; 95%CI: -1.300 to -0.272; P=0.0027; Figure 3A).

Three studies reported body composition data. The meta-analysis showed no significant difference between the WBV group and the control group in muscle mass after intervention (WMD=0.547; 95%CI: -1.104 to 2.199; P=0.5158; Figure 3B). Similarly, pooled analysis of fat mass did not show a difference between the two groups (WMD=0.530; 95%CI: -2.389 to 3.448; P=0.7222; Figure 3C).

### Publication bias

**Figure 4:**
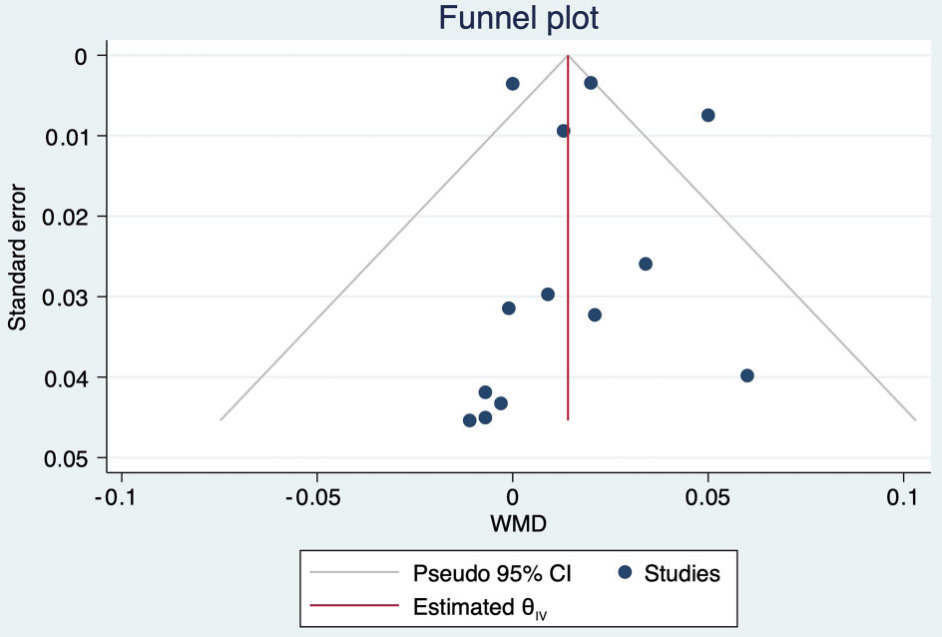
Funnel plot of publication bias of publications on whole body vibration (WBV).

The funnel plot showed that the distribution of studies was relatively symmetrical, suggesting that no obvious evidence of publication bias existed for BMD assessment (P=0.643; Figure 4).

## Discussion

The aim of this review was to evaluate the therapeutic effects of WBV on postmenopausal women with osteoporosis. There were 13 RCTs that met the inclusion criteria and were included in the meta-analysis. The results suggested that WBV was beneficial for improving lumbar spine and femoral neck BMD, as well as decreasing pain scores of postmenopausal women. However, it had no significant effect on the patients' anthropometric measurements.

Previous studies have explored the effectiveness of WBV on bone density in individuals with osteoporosis, but the conclusions still remained controversial. Ruan et al. (28), using a WBV protocol with a frequency of 30 Hz and an amplitude of 5 mm for 6 months, found a 4.3% improvement in lumbar spine BMD and a 3.2% improvement in femoral neck BMD in postmenopausal women. A second study, by Davis et al. (34), compared the effects of two different WBV intensities with walking training in postmenopausal osteoporosis, but the results indicated no significant increases in BMD among the low-intensity group (30-35 Hz, 2 mm), high-intensity group (40-50 Hz, 4 mm), and the control group. The variations in WBV protocols, such as vibration frequency, magnitude, and the administration of WBV, were considered as the main reasons for the inconsistent findings. To investigate the factors which influence the effects of WBV on the BMD of patients with osteoporosis, some meta-analyses have been conducted (35). The study of Oliveira et al. (36) in 2016 reported that a high frequency combined with low WBV magnitude contributed to the increase in the BMD of the lumbar spine. Only postmenopausal women with osteoporosis were enrolled in that study, and the results of meta-analysis supported the short-term (6 months) beneficial effects of WBV on patients' BMD, while its substantial benefits were not observed on the long term (12 months). This could be attributed to the patients' clinical conditions or their physiological adaptations to the mechanical stimuli induced by WBV (29). To date, the mechanism underlying the osteogenic effect of WBV is not fully understood. One hypothesis is that the mechanical vibration can induce microtrauma to bone tissue, stimulating osteoblasts to repair and increase bone density in response to altered loading conditions (37). Another mechanism of the beneficial action of WBV for BMD may be associated with its ability to increase the calcium supply and subsequently stimulate skeletal mineralization (24).

The present study also found that WBV was effective in reducing back pain in osteoporotic elderly women. Previous studies have confirmed that WBV can relax the back muscles and increase their blood flow in patients with chronic back pain (38). Thus, the benefit of WBV for pain relief may result partially from alleviating spasm and ischemia of back muscles, and then by removing fatigue-related substances (39). Additionally, the results revealed that WBV did not improve patients' muscle mass or fat mass. It should be noted that a definite conclusion could not be drawn as limited data (only 2 RCTs) were synthesized and analyzed.

With respect to the quality of the included studies, the PEDro scale classified them as having good methodological quality. Therefore, the pooled results provided robust evidence supporting the application of WBV in postmenopausal women with osteoporosis.

Our findings should be interpreted in the context of several limitations. First, most RCTs had a small sample size (only one study had a sample of over 100 participants), which may weaken the statistical power of the results. Subsequently, some original data could not be directly obtained and thus needed to be estimated, potentially affecting the accuracy of the meta-analysis results. Third, there were differences between studies in terms of WBV protocols and study populations, such as disease duration and the degree of osteoporosis, which could potentially influence the pooled effect of WBV. Finally, due to the limited number of included studies, we were unable to conduct further subgroup analyses.

### Conclusion

WBV showed a potential to provide positive benefits in improving BMD and pain of postmenopausal women with osteoporosis, while no significant improvement was found in terms of body composition.
